# 

**Published:** 1903-11-28

**Authors:** 


### CONTENTS.

**Food Adulteration in 1902** - 143

**Post-Graduation Teaching in London** - 144

**Annotations** - 145

**Notes and News** - 146

**Hospital Clinics and Medical Progress—**

Infective Endocarditis - 147

Ethyl Chloride as a General Anæsthetic - 148

The Treatment of Nephritis - 148

Macroglossia - 149

Operative Treatment of Tuberculous Glands in the Neck - 149

Progress in State Medicine - 150

Progress in Bacteriology - 151

Progress in Disease of Digestive Organs - 152

**New Appliances and Things Medical** - 153

**The Book World of Medicine and Science** - 154

**Modern Sociology** - 155

**Hospital Administration—**

The Future of St. Bartholomew's Hospital - 158

British Institutions for the Care of the Inebriate—V. - 159

**Editor's Letter-Box** - 160

**The Student of Medicine** - 160

*NURSING SECTION.*

**Notes on News from the Nursing World—** Our Christmas Distribution—Queen Alexandra and District Nurses—The Matron of Johannesburg Hospital and Her Nurses—The Liability of Nursing Associations for the Sick Poor—Domestic Economy and Nursing—Opposition to a Residential Club in Melbourne—A Nurse Expected to Dine with a Stableman—Poor law Inspectors and Nursing—Is a Nurse an Inhabitant?—The Nurses of Toronto General Hospital—Nurses' Missionary Union—New Quarters for Huddersfield Infirmary Nurses—Sheffield Guardians and their Superintendent Nurse—Social Gathering - 111-114

**Lectures upon the Nursing of Mental Diseases** 115

**Incidents in the Bombay Fever Wards** - 117

**Presentations** - 118

**New Books on Nursing** - 118

**Everybody's Opinion** - 119

**Appointments** - 120

**Novelties for Nurses** - 120

**Travel Notes and Queries** - 120

**Echoes from the Outside World** - 121

**For Reading to the Sick** - 122

**Notes and Queries** - 122

